# The reliability, validity and responsiveness of the Restless Legs Syndrome Quality of Life questionnaire (RLSQoL) in a trial population

**DOI:** 10.1186/1477-7525-3-79

**Published:** 2005-12-05

**Authors:** Linda Abetz, Robert Arbuckle, Richard P Allen, Elena Mavraki, Jeffrey Kirsch

**Affiliations:** 1 Mapi Values, Adelphi Mill, Bollington, Macclesfield, SK10 5JB, UK; 2 Johns Hopkins Bayview Medical Center, Neurology and Sleep Medicine, Asthma and Allergy Building 1B46b, 5501 Hopkins Bayview Circle, Baltimore, MD 21224, USA; 3 GlaxoSmithKline, Greenford Road, Greenford, Middlesex, UB6 0HE, UK

## Abstract

**Background:**

The aim of this study was to determine the reliability, validity and responsiveness of the Restless Legs Syndrome Quality of Life questionnaire (RLSQoL) in a clinical trial setting.

**Methods:**

Two matching, placebo-controlled, multinational studies assessing the effectiveness and safety of ropinirole for treating moderate-to-severe Restless Legs Syndrome (RLS) formed the basis of this psychometric assessment. Validity and reliability were assessed using baseline data. Responsiveness was determined using longitudinal data collected at baseline and 12 weeks.

**Results:**

A total of 547 subjects formed the baseline validation population; 519 were used for assessing responsiveness (n = 284/263 and 271/248 for both studies, respectively). Construct validity assessment confirmed that an overall life impact score could be calculated. All item-scale correlations were = 0.4, except items 1 (r = 0.36) and 5 (r = 0.35) in one study. Floor and ceiling effects were minimal. Cronbach's alpha values were 0.82 and 0.87, respectively, confirming internal consistency reliability. Correlations with the International Restless Legs Syndrome Study Group's severity rating scale (International Restless Legs Scale; IRLS) were moderate (r = -0.68 and -0.67, respectively; p < 0.0001). The RLSQoL was able to discriminate between levels of sleep problems (p < 0.0001) and between levels of global health status determined by a Clinical Global Impression of severity (CGI-S) (p < 0.0001). Responsiveness was demonstrated by significant differences in overall life impact change scores between CGI improvement levels after 12 weeks (p < 0.0001).

**Conclusion:**

The RLSQoL is a valid, reliable and responsive measure of quality of life for patients with RLS, in a clinical trial setting where group comparisons are anticipated.

## Background

Restless Legs Syndrome (RLS) is a debilitating neurological disorder characterized by a strong urge to move the limbs, usually accompanied by unpleasant sensations, such as creeping, crawling, tingling or pain, particularly when the person lies down or sits for prolonged periods. In most patients, these sensations are felt in the legs, but they may also occur in the arms or trunk [1]. Both the urge to move and these sensations represent the primary symptoms of RLS. Movement brings almost immediate but variable relief from the symptoms, and this relief is maintained as long as the movement continues. If patients ignore the urge to move their legs, there may be intensification of the symptoms until the urge is satisfied, either voluntarily or involuntarily [2]. As a result, RLS can have serious adverse effects on sleep, leaving patients with reduced sleep time and daytime fatigue, reduced concentration and decreased motivation, which in some cases can lead to depression and anxiety [3,4].

The prevalence of RLS in the general population has been reported to range from 5% to 10% [5] with an increasing prevalence with age, and a somewhat higher rate in women than in men [2,6]. There is often a family history of RLS, particularly among patients who present with RLS before 45 years of age [7]. However, primary RLS is a recognized condition that tends to co-occur in the same family. Recently, possible genetic linkages have been established [8-10]. Secondary RLS has been reported to occur in association with a number of conditions, including pregnancy, end-stage renal failure and iron-deficiency anemia [2]. There are also a number of differential diagnoses to be excluded, including leg cramps or paraesthesias, and hypotensive akathisia.

A number of agents are used for the treatment of patients with RLS, including dopaminergic agents and opiates, and treatment varies according to symptom severity and frequency, and the presence or absence of painful symptoms. Although treatments may relieve some or all of the symptoms of RLS, Trenkwalder and colleagues [11] reported that they can also be associated with side effects, including increased symptom severity (augmentation) in the long term. Both the disorder and its treatment may, therefore, have an impact on patients' quality of life. For this reason, the Restless Legs Syndrome Quality of Life questionnaire (RLSQoL), a patient-reported measure of quality of life specific to patients with RLS, was developed.

The RLSQoL has been previously validated in an independent study [12]. Results from that study indicated the reliability and validity of the RLSQoL. However, the study was limited by its relatively small sample size and by the lack of a full assessment of the responsiveness of the RLSQoL to change over time (all subjects maintained their normal treatment regimen throughout the 2-week study, yielding few changes). The aim of the present study, therefore, was to assess the reliability, validity and responsiveness of the RLSQoL in a larger sample size, over a longer period of time (12 weeks) and with treatment intervention. Thus, this study is based on the patient populations of two recently completed, matching, phase-III, multicentre, randomized, double-blind, placebo-controlled studies assessing the efficacy and tolerability of ropinirole, a dopamine agonist, for the treatment of patients with RLS (Therapy with Ropinirole: Efficacy And Tolerability in RLS [TREAT RLS] 1 and 2). Findings from both studies have been published in full elsewhere [11,13].

The primary endpoint in both studies was change in total score of the International Restless Legs Syndrome Study Group's severity rating scale (International Restless Legs Scale, IRLS) [14,15], a clinician-administered report of patient symptom severity. However, the RLSQoL was included as a secondary endpoint. Although the findings of the two separate trials are being presented together in this paper, it should be noted that the analysis for each study was conducted separately.

## Methods

### Patient populations

Patients were eligible for inclusion in TREAT RLS 1 and 2 if they were at least 18 years of age, had a baseline IRLS total score of ≥ 15 (on a scale of 0 to 40, indicating moderate-to-severe RLS) and had moderate-to-severe frequency of RLS (experienced at least 15 nights with symptoms of RLS in the previous month or, if receiving treatment, had symptoms of this frequency prior to treatment). Patients were excluded from the study if they had any other movement or primary sleep disorder, if they required daytime treatment for RLS, if they were experiencing augmentation or end-of-dose rebound, or if they had secondary RLS associated with end-stage renal disease, iron-deficiency anemia or pregnancy. Patients were also excluded if they had a history of alcohol or drug abuse, previous intolerance to dopamine agonists, or were suffering from other clinically relevant conditions affecting assessments.

All patients gave written, informed consent before entering the studies, which was done according to the principles of the 1996 amendment of the Declaration of Helsinki and approved by local ethics committees.

### Study design

Both studies were conducted in a matching double-blind, randomized, placebo-controlled fashion. Patients were recruited from hospitals, sleep centres and neurology clinics in 10 European countries in TREAT RLS 1 (Austria, Belgium, France, Germany, Italy, Netherlands, Norway, Spain, Sweden and the UK) and in six countries around the world in TREAT RLS 2 (Australia, Canada, Germany, Norway, the UK and the USA). After a wash-out phase of generally 5 half-lives or 7 consecutive nights medication-free, whichever was the longer period, patients were randomized to receive once-daily treatment with either ropinirole or placebo for 12 weeks. Other published articles report the study design in greater detail [11,13].

The primary endpoint in both studies was the change in the IRLS total score; secondary endpoints included the RLSQoL overall life impact score, the Medical Outcomes Study Sleep Problems Index II (MOS Sleep Scale) score and Clinical Global Impression (CGI) 'Improvement' (CGI-I) and 'Severity of Illness' (CGI-S) scores.

### Outcome measures used in psychometric analysis

#### RLSQoL

The RLSQoL is a validated questionnaire consisting of 18 items, 13 of which are scored on a 5-point scale, the remainder being recorded as either a numerical value or a dichotomous response [12]. Ten of the items contribute to a single summary score, the overall life impact score, while the remaining eight items concern employment (one question), sexual interest (two questions) and work (five questions), and are summarized individually. Details of the questionnaire and scoring can be found in the appendix (see additional file 1). Higher scores on the RLSQoL overall life impact score indicate a better quality of life. Patients were asked to complete the RLSQoL at baseline and at weeks 8 and 12 of the treatment phase, or at time of withdrawal for patients who discontinued the studies prematurely. A full listing of the items of the RLSQoL is provided elsewhere [12]. The RLSQoL is available on request from Mapi Values.

#### MOS Sleep Scale

The MOS Sleep Scale is a self-administered scale measuring specific aspects of sleep (problems with sleep disturbance [initiation and maintenance], adequacy, somnolence, quantity, respiratory impairments and snoring) and is reliable and valid in the general US population [16]. It was designed for use in patients who may have varying co-morbidities, and hence is appropriate for a medically diverse patient population. The frequency with which each problem has been experienced during the previous 4 weeks is rated on a 6-point scale ranging from 'none of the time' to 'all of the time', except sleep quantity, which is reported in hours. All scores are transformed linearly to range from 0 to 100, again with the exception of the sleep quantity subscale, which is scored in hours. Higher scores indicate more of the attribute implied by the scale name (e.g. more sleep disturbance, more adequate sleep, greater sleep quantity). Patients were asked to complete the MOS Sleep Scale at baseline and at weeks 8 and 12 of the treatment phase, or at the time of withdrawal for patients who discontinued the study prematurely. The psychometric properties of the MOS Sleep Scale have been found to be satisfactory, both by Hayes and colleagues [17], and within each of the two clinical trial populations used in this study, as recently reported at the 16th Annual Scientific Meeting of the British Sleep Society, Cambridge, UK, 19–21 September 2004.

#### CGI

The CGI consists of three modules, the CGI 'Improvement' (CGI-I), the CGI 'Severity of Illness' (CGI-S), and the CGI 'Efficacy Index', and has been in use for nearly 3 decades [18]. In the present two studies, only the first two modules were used as outcome measures, although the CGI 'Efficacy Index' was used by the investigators to guide titration of the study medication. The CGI-I and CGI-S modules were assessed by the investigator, based on all information available at the time of rating. Both modules were rated on a scale of 0–7, where 0 refers to patients who were not assessed, 1 indicates 'very much improved' and 7 indicates 'very much worse'. Changes in the proportions of patients with scores of 'much improved' or 'very much improved' were identified as two key secondary endpoints. Both the CGI-I and CGI-S were assessed by the investigators at day 2 and weeks 1, 2, 3, 4, 5, 6, 7, 8 and 12 of the treatment phase, or at the time of withdrawal in patients who discontinued the study prematurely. The CGI-S was also assessed at baseline.

#### IRLS

The IRLS was developed and validated by the International Restless Legs Syndrome Study Group [14,15]. Subsequent validation studies were conducted for the IRLS using TREAT RLS 1 and TREAT RLS 2 data, and results confirmed the reliability, validity and responsiveness of the IRLS [19]. The IRLS consists of 10 questions concerning the patient's symptoms and the impact of these symptoms on daily activities and mood. Responses range from 0 to 4, with 0 representing the absence of a problem and 4 representing a very severe problem.

The IRLS was completed at the baseline visit, at day 2 and weeks 1, 2, 3, 4, 5, 6, 7, 8 and 12 of the treatment phase and at the follow-up assessment, or at the time of withdrawal for patients who withdrew prematurely.

### Analysis

#### Study populations

In both studies, the intention-to-treat (ITT) population included all randomized patients who received at least one dose of study medication and who had at least one post-baseline efficacy measurement. Patients from the ITT population who had an evaluable RLSQoL (i.e. at least eight non-missing items among items 1–5, 7–10 and 13, as specified by the developer of the questionnaire) were included in the RLSQoL baseline validation population, which was used for all psychometric analyses of the questionnaire, except responsiveness. Patients included in the baseline validation population who also had an evaluable RLSQoL at the 12-week post-baseline visit were included in the longitudinal validation population, which was used for analysis of the responsiveness to change over time of the RLSQoL. All tests were performed on the total population samples, blinded to treatment status.

#### Psychometric validation analyses

The RLSQoL was assessed for the following psychometric properties: item convergent validity (item-scale correlations of ≥ 0.4) [20], floor and ceiling effects (the percentage scoring the lowest and highest possible scores), internal consistency reliability (Cronbach's alpha ≥ 0.7), concurrent validity, known groups validity, clinical validity and responsiveness.

Assessment of concurrent validity consists of examining the association between the measure being validated, and other well-validated measures, assessing similar constructs. In this instance, concurrent validity was evaluated by assessing correlations between the RLSQoL overall life impact score and the IRLS total score, a clinician-administered patient report of RLS symptom severity. As the RLSQoL has some items related to sleep and somnolence, the correlations of the RLSQoL overall life impact score with the MOS Sleep Scale Sleep Problems Index II was also assessed. Correlations of ≥ 0.40 were considered sufficient evidence of concurrent validity.

The known groups validity of the RLSQoL was assessed by describing and comparing RLSQoL overall life impact scores at baseline among groups of patients with mild, moderate and severe sleep problems, as defined by taking tertile scores for the Sleep Problems Index II of the MOS Sleep Scale. Tertile scores were used because no clinical cut-offs are available for the MOS Sleep Scale. Taking tertile scores involves dividing a normally distributed population into three groups of as close as possible to 33% of patients in each group [21]. Scores of 0–41, 42–56 and 57–100 were considered mild, moderate and severe, respectively. The hypothesis was that patients with more severe sleep problems would have worse quality of life, indicated by lower RLSQoL overall life impact scores.

The clinical validity of the RLSQoL was assessed in two ways. First, the correlation between the RLSQoL overall life impact score and the CGI-S score at baseline was assessed, with a correlation of ≥ 0.40 considered sufficient to confirm validity. Second, RLSQoL overall life impact scores at baseline were compared between severity subgroups defined by dividing the patients into three groups on the basis of their CGI-S scores. The groups were comprised as follows: (1) normal, not at all ill or borderline ill (CGI-S scores 1–2); (2) mild, moderately or markedly ill (CGI-S scores 3–5); (3) severely ill, or among the most extremely ill patients (CGI-S scores 6–7). Patients with a CGI-S score of 0 ('not assessed') were excluded from this analysis. The hypothesis was that for worse clinician-rated overall health status, RLSQoL overall life impact scores would also be worse.

The responsiveness of the RLSQoL to change over time was assessed in two ways. First by examining the correlations between the change in RLSQoL overall life impact scores (between baseline and weeks 8 and 12) and CGI-I scores (1–7) at weeks 8 and 12. Patients with a CGI-I score of 0 ('not assessed') were excluded from the analysis. Second, RLSQoL overall life impact change scores (subtracting baseline from week 12 assessments) were compared among patients defined as improved, unchanged and worsened at week 12, on the basis of their CGI-I scores. Patients with a CGI-I score of 0 ('not assessed') were excluded from the analysis. The effect size (ES) was used as a measure of the change in RLSQoL scores within each CGI-I group. ESs were calculated by dividing the change in mean RLSQoL overall life impact scores (from baseline to week 12) by the standard deviation of mean scores at baseline. The ES has been recommended in the literature as an appropriate benchmark for evaluating the magnitude and meaning of change in health status measures [22].

Cohen and colleagues defined effect sizes of 0.2, 0.5 and 0.8 as small, moderate and large, respectively [23]. We adopted Guyatt et al's guidance that size effects can be described as small, moderate or large when results are in the range of these parameters [24].

### Statistics

No adjustments for multiplicity were performed. The Type 1 criterion was 0.05, and all hypothesis tests were two-sided. As tests of homeoscedasticity (equality of dispersion) and normality did not find the analysis data to be normally distributed, non-parametric tests were used for all comparisons. Therefore, Pearson's correlation coefficient was used for all correlations evaluated, the Kruskall-Wallis test was used for comparisons between more than two groups, the Mann-Whitney-Wilcoxon test was used for comparisons between pairs of groups and the Wilcoxon signed rank test was used for comparing two points in time within groups.

## Results

### Patient populations

TREAT RLS 1 included 284 patients in the baseline validation population, and 229 patients in the week 12 longitudinal validation (responsiveness) population. TREAT RLS 2 included 263 patients in the baseline population and 207 patients in the week 12 longitudinal validation population. Baseline patient characteristics for each study are shown in Table 1. In both studies, the majority of patients were categorized as moderately, markedly or severely ill. The mean age of patients in each study was 55 years and approximately two-thirds of patients in each study were women (63.0% and 59.7%, respectively). There was a slight difference in the mean age of onset in TREAT RLS 1 and TREAT RLS 2: 38 and 35 years, respectively.

### Psychometric validation

#### Missing data

Missing data for TREAT RLS 1 and 2 are summarized in Table 2. In TREAT RLS 1, missing data for each of the items of the RLSQoL ranged from 0% missing data for items 1, 2, 4, 7, 8 and 10 to 3.52% (n = 10) for item 5 ('In the past 4 weeks how often were you late for work or your first appointment due to RLS?'). In TREAT RLS 2, missing data levels ranged from 0% missing data for items 1, 4, 7, 8, 9 and 13 to 1.52% for item 5. Therefore, missing data at the item level were not problematic in either study.

#### Factor analysis

The current scoring of the RLSQoL, devised by the developers of the questionnaire, suggests calculating a summary score, i.e. the overall life impact score.

A commonly used criterion for calculating a summary score is that the cumulative variance of the first factor in a principal component analysis of ≥ 0.40, although lower values are sometimes also used (e.g. 0.3). In the present two studies, principal component analysis resulted in a cumulative variance of 0.39 in TREAT RLS 1, and 0.46 in TREAT RLS 2. Given that the criterion was surpassed in TREAT RLS 2, and very narrowly missed in TREAT RLS 1, it was considered acceptable for the overall life impact score to be calculated.

The results of the construct validity analysis demonstrated excellent reliability and construct validity for the RLSQoL, as summarized in Table 3.

#### Item convergent validity

The criterion for item convergent validity (item-scale correlations ≥ 0.40) was satisfied by all items in TREAT RLS 2. In TREAT RLS 1, all except two items met the criterion for item convergent validity. However, both items only narrowly missed the 0.40 threshold with correlations of 0.36 (item 1: 'In the past 4 weeks how distressing to you were your restless legs?') and 0.35 (item 5: 'In the past 4 weeks how often were you late for work or your first appointments of the day due to restless legs?'), respectively.

#### Internal consistency reliability

Cronbach's alpha coefficients for the RLSQoL overall life impact score were 0.82 and 0.87 in TREAT RLS 1 and 2, respectively, indicating satisfactory internal consistency reliability for the RLSQoL in both trials.

#### Floor and ceiling effects for the RLSQoL overall life impact score

For the RLSQoL overall life impact score, in TREAT RLS 1, 0.35% (n = 1) of patients scored at floor, 0% scored at ceiling. In TREAT RLS 2, 0% of patients scored at floor and 0.38% (n = 1) scored at ceiling. Therefore, there were no significant floor or ceiling effects for the RLSQoL overall impact score in either study.

#### Concurrent validity

Correlations of the RLSQoL overall life impact score with the concurrent measures are provided in Table 4. All correlations were above the 0.40 standard set for concurrent validity. In both TREAT RLS 1 and 2, the RLSQoL overall life impact score was moderately correlated at a statistically significant level with both the IRLS total score (r = -0.68 and r = -0.67, respectively) and the Sleep Problems Index II (r = 0.59 and r = 0.60, respectively). These results confirm the concurrent validity of the RLSQoL.

#### Known groups validity

**Figure 1:**
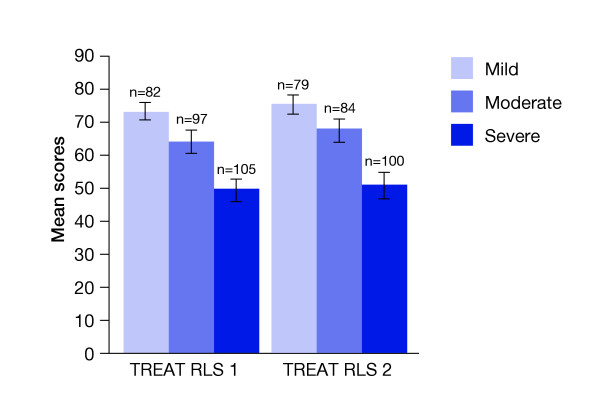
**Known groups validity**. RLSQoL overall life impact scores by mild, moderate and severe sleep problems. p < 0.0001 for comparisons of RLSQoL overall life impact scores among sleep problems severity groups defined by taking tertile scores for the Sleep Problems Index II in TREAT RLS 1 and 2 (Kruskall-Wallis test). RLSQoL = RLS Quality of Life questionnaire.

In both TREAT RLS 1 and 2, the results indicate that the RLSQoL overall life impact scores distinguished between groups of mild, moderate and severe sleep problems at a statistically significant level (p < 0.0001); patients with more severe sleep problems had lower RLSQoL overall life impact scores (poorer quality of life) (Figure 1). Mean overall life impact scores for mild, moderate and severe groups were 73.04, 63.68 and 49.26, respectively, in TREAT RLS 1, and 74.93, 67.44, and 50.54, respectively, in TREAT RLS 2. These results indicate the 'known groups' or discriminative validity of the RLSQoL.

#### Clinical validity

**Figure 2:**
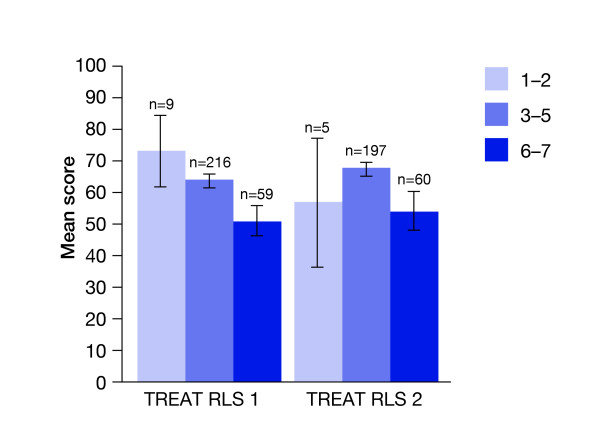
**Clinical validity**. Comparison of RLSQoL overall life impact scores at baseline among CGI-S groups. CGI-S subgroups 1–2 include normal, not at all ill and borderline ill patients; subgroups 3–5 include mild, moderate, and markedly ill patients; and subgroups 6–7 include severely ill, and the most extremely ill patients. p < 0.0003 (Kruskall-Wallis test comparing all three subgroups) and p < 0.000.1 (Mann-Whitney-Wilcoxon test comparing subgroups 3–5 with 6–7) for comparisons of RLSQoL overall life impact scores among collapsed CGI-S subgroups in both TREAT RLS 1 and 2; p = not significant (Mann-Whitney-Wilcoxon test comparing subgroups 1–2 with 3–5). RLSQoL = RLS Quality of Life questionnaire. CGI-S = Clinical Global Impression 'Severity of Illness'.

First, clinical validity was assessed by examining the correlation between RLSQoL overall life impact scores and CGI-S scores. The correlation between RLSQoL overall life impact scores and CGI-S scores was moderate (r = -0.42, p < 0.0001) in TREAT RLS 1, and low but statistically significant (r = -0.33, p < 0.0001) in TREAT RLS 2. In addition, statistically significant differences in RLSQoL overall life impact scores were observed between the three CGI-S subgroups (p < 0.0003) in both TREAT RLS 1 and 2 (Figure 2). Impairment in quality of life due to RLS was greater for groups with worse clinician-rated RLS severity. The differences between pairs of adjacent groups were assessed further using the Mann-Whitney-Wilcoxon test. However, in both studies, the subgroup of patients with CGI-S scores of 1–2 (very mild) was very small, most likely as a consequence of the inclusion criteria for the studies (moderate/severe). Comparisons with this mild group should, therefore, be interpreted with caution. Nevertheless, statistically significant differences in RLSQoL overall life impact scores between the two larger CGI-S subgroups, 3–5 and 6–7, were observed in both studies (p < 0.0001 in both). These findings provide evidence that the RLSQoL is clinically valid in this clinical trial population.

#### Responsiveness to change over time

When assessing responsiveness, trends for the correlations, change scores and effect sizes were similar at week 8 and week 12. (For brevity, only week 12 results are reported here; week 8 results are available on request.) First, the responsiveness of the RLSQoL to change over time was suggested by moderate and statistically significant correlations of RLSQoL overall life impact change scores with CGI-I scores (r = -0.51, p < 0.0001 in both studies). Investigating this relationship further, statistically significant differences in RLSQoL overall life impact change scores (from baseline to week 12) were observed between groups stratified according to CGI-I scores, in both studies (Tables 5 and 6; p < 0.0001). In both studies, there was a step-wise increase in effect sizes for the no change, and minimally, much and very much improved groups, indicating greater improvements in RLSQoL scores for the more improved CGI-I groups compared with the less improved and no change groups. Effect sizes indicated large improvements in overall life impact scores in the 'very much improved' (ES = 1.51, in both studies), and 'much improved' groups (ES = 1.15 and 1.00 in TREAT RLS 1 and 2, respectively), large and moderate improvements in the 'minimally improved' groups (ES = 0.74 and 0.54, respectively) and moderate or small improvements in the 'no change' group (ES = 0.37 and 0.31, respectively). In TREAT RLS 1 and 2, patient numbers were very small in the 'minimally worse' (5 and 6, respectively), 'much worse' (9 and 0, respectively) and 'very much worse' (0 and 1, respectively) CGI-I groups; therefore, comparisons with these groups cannot be evaluated.

These findings indicate that the RLSQoL is responsive to clinician-rated changes in health status.

## Discussion

Based on the results of this psychometric evaluation, the RLSQoL has been found to be reliable, valid and responsive to change in both TREAT RLS 1 and 2. Although the psychometric validity of the RLSQoL has been demonstrated previously [12], due to the evolutionary nature of validation it is important to confirm the psychometrics of a questionnaire when used in different settings and with different populations. The results provide evidence of the psychometric integrity of the RLSQoL within the RLS populations studied, and support its use in patients with RLS, particularly in a clinical trial setting.

The factor analysis results support the calculation of a summary score, the overall life impact score, based on 10 of the 18 items. Although the factor analysis results in TREAT RLS 1 gave a cumulative variance of only 0.39, narrowly missing the 0.40 criterion for calculating a summary score, published research suggests that the cut-off point of 0.40 is 'arbitrary' [25]. Therefore, given that the criterion was surpassed in TREAT RLS 2 (with cumulative variance of 0.46), and only narrowly missed in TREAT RLS 1, it is still considered appropriate to calculate a summary score. This position is further supported by the satisfactory psychometric validation results, and evidence from a previous independent validation study which also concluded that calculating the overall life impact score was valid and appropriate [12].

Both the original validation study and the present research have focused on evaluating the psychometric validity of the overall life impact score: the validity of potential subscales could be investigated in future studies [12]. In addition, sample sizes did not permit factor analyses to be conducted for each country separately; further research could examine differences in scale structure by country or within different age groups.

Taking the findings of both studies together, item convergent validity results were satisfactory. Two items did narrowly miss the criterion (item-scale correlations ≥ 0.40) in TREAT RLS 1. However, given that they were only slightly below the threshold in TREAT RLS 1, and met the criterion in TREAT RLS 2, this was of little concern. The internal consistency reliability of items in the RLSQoL overall life impact score was acceptable in both studies, with Cronbach's coefficients exceeding the accepted standard (≥ 0.70). There were no significant floor or ceiling effects in either study.

In the assessment of concurrent validity, the moderate correlations between the RLSQoL overall life impact score and the IRLS total score indicate that there is some overlap between the RLSQoL and the IRLS, but not so much overlap as to suggest redundancy (> 0.9). The IRLS and RLSQoL assess different concepts (severity and quality of life, respectively) and therefore it is not surprising that the overlap is not greater.

Correlation between the RLSQoL overall life impact scale and the MOS Sleep Scale Sleep Problems Index II was also moderate in both studies, providing evidence that sleep problems do have an impact on quality of life. These results confirm the concurrent validity of the RLSQoL.

In both studies, the RLSQoL overall life impact score was able to distinguish between patients with mild, moderate and severe sleep problems. The results indicate that patients with more severe sleep problems also had lower RLSQoL overall life impact scores (poorer quality of life), thus demonstrating the known groups validity of the RLSQoL overall life impact score.

The findings indicated that as the CGI-S scores increased, RLSQoL overall life impact scores worsened. The fact that the correlation between CGI-S and the RLSQoL overall life impact scores was only low to moderate is unsurprising, given that correlations between doctors' ratings of severity and patient reports of severity are often low to moderate [26,27].

In addition, the RLSQoL overall impact scores were able to distinguish between the three clinician-rated severity groups. The sample sizes in the 'mild' groups were very small, and the results for these groups should, therefore, be interpreted with caution. However, RLSQoL overall life impact scores for the 'moderate' CGI-S groups were statistically significantly different from those for the 'severe' CGI-S groups in both studies. Of note, although these studies did not assess 'mild' RLS patients, the original validation did assess this group (in addition to patients with moderate and severe RLS). The combination of these results indicates the clinical validity of the RLSQoL in mild, moderate and severe groups [12].

The responsiveness of the RLSQoL to change over time was confirmed by comparing change scores from baseline to week 12 with clinicians' perceived changes (CGI-I). Correlation of changes in RLSQoL overall life impact score with CGI-I scores was moderate and statistically significant in both studies. RLSQoL overall life impact change scores were able to distinguish between CGI-I subgroups at a statistically significant level in both studies. RLSQoL scores were improved in patients rated by their clinicians as 'improved', as well as in those patients rated by their clinicians as 'unchanged'. However, effect sizes indicated the improvements were consistently 'large' in those patients rated as 'improved' but only small or moderate in those rated as 'unchanged'. Sample sizes for the worsened groups were small and results for these groups should be interpreted with caution. Improvements in the 'no change' CGI group suggest the potential presence of the 'Hawthorne effect'; that is, a response shift most probably due to positive psychosocial effects of participating in a clinical trial, regardless of the specific nature of any intervention [26].

The sensitivity of the RLSQoL to worsening RLS severity could not be fully evaluated in this study owing to the small sample size of worsening patients, and should be investigated further in a larger sample of worsening patients at the first opportunity. Therefore, it cannot be concluded that the RLSQoL is responsive to worsening until further research is conducted. Furthermore, due to the inclusion criteria for the trials, there were very few patients who were rated by their clinician as being 'normal, not at all ill', 'borderline ill' or 'mildly ill' at baseline (1.27%, 1.27% and 8.18%, respectively, from the CGI-S at baseline). Therefore, further study of the performance of the responsiveness of the RLSQoL in patients with 'mild' RLS is also warranted.

Finally, the focus of this research was on group comparisons; as a result, additional research is warranted to evaluate the usefulness of the RLSQoL in clinical practice to assess patients individually.

## Conclusion

In conclusion, the RLSQoL is reliable, valid and responsive to improvements in patients with RLS, in a clinical trial setting. The RLSQoL is short, takes 10 minutes to complete, and covers those aspects of life most impacted by RLS. Other, more generic, questionnaires will not be as relevant to patients and therefore will not be as clinically relevant.

## Authors' contributions

LA, EM, JK, and RPA all participated in the design of the study. LA and RA wrote the analysis plan and supervised the analysis. All authors were involved in the interpretation of the data. LA, RA, and JK were involved in drafting the article, which was then revised following critical review by EM and RPA. All authors read and approved the final manuscript.

## Supplementary Material

Additional File 1 **Appendix: RLS Quality of Life Questionnaire** Click here for file

## Figures and Tables

**Figure 3:**
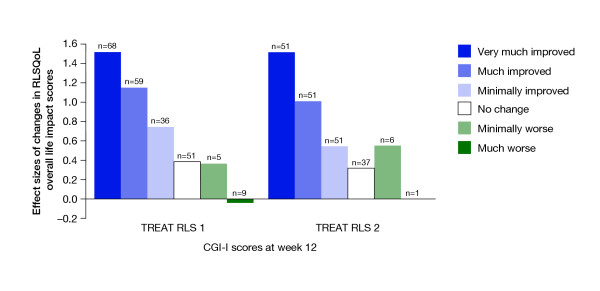
**Responsiveness**. Effect sizes as a measure of the change in RLSQoL overall life impact change scores between baseline and week 12, by CGI-I scores at week 12. p < 0.0001 for comparisons of the change in RLSQoL overall life impact scores among CGI-I groups in both TREAT RLS 1 and 2 (Kruskall-Wallis test). The sample size of n = 1 in the 'worsened' group in TREAT RLS 2 meant that effect sizes could not be calculated. RLSQoL = RLS Quality of Life questionnaire. CGI-I = Clinical Global Impression 'Improvement'.

**Table 1:** Patient characteristics at baseline for TREAT RLS 1 (n = 284) and TREAT RLS 2 (n = 263)

	**TREAT RLS 1**	**TREAT RLS 2**
Age, years		
Mean (SD)	55.1 (11.2)	55.4 (11.1)
Range	28.0–78.0	29.0–79.0
Sex, % (n)		
Men	37.0 (105)	40.3 (106)
Women	63.0 (179)	59.7 (157)
Work status, % (n)		
Full-time employment	36.3 (103)	43.4 (114)
Part-time employment	15.1 (43)	12.6 (33)
Volunteer/unpaid work	2.5 (7)	1.9 (5)
Unemployed due to RLS	1.1 (3)	0.8 (2)
Unemployed due to other (non-RLS) symptoms	3.2 (9)	0.4 (1)
Unemployed for other reasons	8.5 (24)	4.2 (11)
Retired	22.2 (63)	29.3 (77)
Homemaker	11.3 (32)	7.6 (20)
CGI severity of illness, % (n)		
Missing data	0 (0)	0.4 (1)
Normal, not at all ill	2.1 (6)	0.4 (1)
Borderline ill	1.1 (3)	1.5 (4)
Mildly ill	9.2 (26)	7.2 (19)
Moderately ill	34.9 (99)	36.5 (96)
Markedly ill	32.0 (91)	31.2 (82)
Severely ill	19.0 (54)	20.2 (53)
Among the most extremely ill patients	1.8 (5)	2.7 (7)
Item 6 of the IRLS: 'How severe was your RLS as a whole?', % (n)		
None	0 (0)	0 (0)
Mild	2.1 (6)	2.3 (6)
Moderate	34.9 (99)	36.9 (97)
Severe	43.7 (124)	44.9 (118)
Very severe	19.4 (55)	16.0 (42)
Item 7 of the IRLS: 'How often did you get RLS symptoms?', % (n)		
Rarely (< 1 day a week)	0 (0)	0 (0)
Occasionally (1 day a week)	1.4 (4)	0.4 (1)
Sometimes (2–3 days a week)	10.6(30)	9.1 (24)
Often (4–5 days a week)	25.4 (72)	32.7 (86)
Very often (6–7 days a week)	62.7 (178)	57.8 (152)
Age of onset of symptoms, years Mean (SD)	38.2 (16.8)	34.6 (17.3)

CGI = Clinical Global Impression. IRLS = International Restless Legs Scale. SD = standard deviation.

**Table 2:** Missing data for the RLSQoL items in TREAT RLS 1 and 2

**RLSQoL item**		**TREAT RLS 1 % (n)**	**TREAT RLS 2 % (n)**
1	In the past 4 weeks how distressing to you were your restless legs?	0 (0)	0 (0)
2	How often in the past 4 weeks did RLS disrupt your routine evening activities?	0 (0)	0.38 (1)
3	How often in the past 4 weeks did RLS keep you from attending your evening social activities?	1.06 (3)	0.76 (2)
4	In the past 4 weeks how much trouble did you have getting up in the morning due to restless legs?	0 (0)	0 (0)
5	In the past 4 weeks how often were you late for work or your first appointments of the day due to RLS?	3.52 (10)	1.52 (4)
7	How often in the past 4 weeks did you have trouble concentrating in the afternoon?	0 (0)	0 (0)
8	How often in the past 4 weeks did you have trouble concentrating in the evening?	0 (0)	0 (0)
9	In the past 4 weeks how much was your ability to make good decisions affected by sleep problems?	0.35 (1)	0 (0)
10	How often in the past 4 weeks would you have avoided travelling when the trip would have lasted more than 2 hours?	0 (0)	0.38 (1)
13	In the past 4 weeks how much did your restless legs disturb your ability to carry out daily activities, for example carrying out a satisfactory family, home, social, school or work life?	0.35 (1)	0 (0)

**Table 3:** Construct validity and reliability of the RLSQoL overall life impact score

	**TREAT RLS 1**	**TREAT RLS 2**
Item-convergent validity, % items with item-scale correlation ≥ 0.40 (coefficient range)	85% (0.35–0.66)	100% (0.46–0.70)
Floor/ceiling effects (% of respondents with minimum/maximum scale scores)	0.35%/0.00%	0.00%/0.38%
Internal consistency reliability, Cronbach's alpha coefficient (satisfactory if ≥ 0.70)	0.82	0.87

RLSQoL = Restless Legs Syndrome Quality of Life questionnaire.

**Table 4:** Concurrent validity: correlations between the RLSQoL overall life impact score and the scores for the IRLS total score and the MOS Sleep Problems Index II (TREAT RLS 1 and 2)^a^

**RLSQoL overall life impact score**		**IRLS total score**	**MOS Sleep Scale Sleep Problems Index II**
TREAT RLS 1	Spearman correlation coefficient	-0.68	-0.59
	p value	< 0.0001	< 0.0001
	Number of patients	284	284
TREAT RLS 2	Spearman correlation coefficient	-0.67	-0.60
	p value	< 0.0001	< 0.0001
	Number of patients	263	263

IRLS = International Restless Legs Scale. RLSQoL = Restless Legs Syndrome Quality of Life questionnaire. MOS = Medical Outcomes Study.

^a ^Satisfactory if correlation ≥ 0.40.

**Table 5:** Changes in RLSQoL overall life impact scores from baseline to week 12: effect sizes and a comparison between CGI-I levels 1–7 (TREAT RLS 1)

	**n**	**Baseline mean**	**Baseline SD (total)**	**Week 12 mean**	**Mean change**	**Effect size**	**Kruskal-Wallis test (p value)**
Very much improved	68	64.33	18.25	91.95	27.62	1.51	0.0001
Much improved	59	62.41	18.25	83.47	21.06	1.15	
Minimally improved	36	57.84	18.25	71.38	13.54	0.74	
No change	51	58.65	18.25	65.41	6.76	0.37	
Minimally worse	5	76.13	18.25	82.61	6.48	0.36	
Much worse	9	58.33	18.25	57.53	-0.8	-0.04	

p < 0.0001 for comparisons of the change in RLSQoL overall life impact scores among CGI-I groups in TREAT RLS 1 (Kruskall-Wallis test). There were no patients included in the CGI-I 'very much worse' category. Effect sizeswere calculated by dividing the change in mean score (from baseline to week 12) by the standard deviation of the mean score at baseline. RLSQoL = RLS Quality of Life questionnaire. CGI-I = Clinical Global Impression 'Improvement'.

**Table 6:** Changes in RLSQoL overall life impact scores from baseline to week 12: effect sizes and a comparison between CGI-I levels 1–7 (TREAT RLS 2)

	**n**	**Baseline mean**	**Baseline SD (total)**	**Week 12 mean**	**Mean change**	**Effect size**	**Kruskal-Wallis test (p value)**
Very much improved	74	63.78	18.83	92.15	28.47	1.51	0.0001
Much improved	43	59.19	18.83	77.94	18.75	1.00	
Minimally improved	45	66.72	18.83	76.94	10.22	0.54	
No change	37	65.48	18.83	71.39	5.91	0.31	
Minimally worse	6	60.83	18.83	70.83	10.00	0.53	
Very much worse	1	80.00	18.83	85.00	5.00		

p < 0.0001 for comparisons of the change in RLSQoL overall life impact scores among CGI-I groups in TREAT RLS 2 (Kruskall-Wallis test). There were no patients included in the CGI-I 'much worse' category. The 'very much worse' group consisted of a single patient and therefore further statistical tests were not performed for this group. Effect sizes were calculated by dividing the change in mean score (from baseline to week 12) by the standard deviation of the mean score at baseline.

RLSQoL = RLS Quality of Life questionnaire. CGI-I = Clinical Global Impression 'Improvement'.

## References

[B1] Allen RP, Picchietti D, Hening WA, Trenkwalder C, Walters AS, Montplaisir J (2003). Restless legs syndrome: diagnostic criteria, special considerations, and epidemiology. A report from the restless legs syndrome diagnosis and epidemiology workshop at the national institutes of health. Sleep Med.

[B2] Earley CP (2003). Restless legs syndrome. N Engl J Med.

[B3] Rothdach AJ, Trenkwalder C, Haberstock J, Keil U, Berger K (2000). Prevalence and risk factors of RLS in an elderly population: the MEMO study. Memory and Morbidity in Augsburg Elderly. Neurology.

[B4] Allen RP, Earley CJ (2001). Validation of the Johns Hopkins Restless Legs Severity Scale (JHRLSS). Sleep Med.

[B5] Hening W, Walters AS, Allen RP, Montplaisir J, Myers A, Ferini-Strambi L (2004). Impact, diagnosis and treatment of restless legs syndrome (RLS) in a primary care population: the REST (RLS Epidemiology, Symptoms, and Treatment) Primary Care study. Sleep Med.

[B6] Allen RP, Earley CJ (2001). Restless legs syndrome: a review of clinical and pathophysiologic features. J Clin Neurophysiol.

[B7] Allen RP, La Buda MC, Becker P, Earley CJ (2002). Family history study of the restless legs syndrome. Sleep Med.

[B8] Desautels A, Turecki G, Montplaisir J, Sequeira A, Verner A, Rouleau GA (2001). Identification of a major susceptibility locus for restless legs syndrome on chromosome 12q. Am J Hum Genet.

[B9] Chen JT, Garcia PA, Alldredge BK (2003). Zonisamide-induced restless legs syndrome. Neurology.

[B10] Bonati MT, Ferini-Strambi L, Aridon P, Oldani A, Zucconi M, Casari G (2003). Autosomal dominant restless legs syndrome maps on chromosome 14q. Brain.

[B11] Trenkwalder C, Garcia-Borreguero D, Montagna P, Lainey E, de Weerd AW, Tidswell P, Saletu-Zyhlarz G, Telstad W, Ferini-Strambi L (2004). Ropinirole in the treatment of restless legs syndrome: results from the TREAT RLS 1 study, a 12-week, randomised, placebo controlled study in 10 European countries. J Neurol Neurosurg Psychiatry.

[B12] Abetz L, Vallow S, Kirsch J, Allen R, Washburn T, Earley C (2005). Validation of the Restless Legs Syndrome Quality of Life Questionnaire. Value Health.

[B13] Walters AS, Ondo WG, Dreykluft T, Grunstein R, Lee D, Sethi K, on behalf of the TREAT RLS 2 Study Group (2004). Ropinirole is effective in the treatment of restless legs syndrome: TREAT RLS 2: a 12-week, double-blind, randomized, parallel-group, placebo-controlled study. Mov Disord.

[B14] Allen RP, Kushida CA, Atkinson MJ (2003). RLS QoL Consortium. Factor analysis of the International Restless Legs Syndrome Study Group's scale for restless legs severity. Sleep Med.

[B15] Walters AS, LeBrocq C, Dhar A, Hening W, Rosen R, Allen RP, Trenkwalder C, on behalf of the International Restless Legs Syndrome Study Group (2003). Validation of the International Restless Legs Syndrome Study Group Rating Scale for restless legs syndrome. Sleep Med.

[B16] Hays RD, Stewart AL, Stewart AL, Ware JE Jr (1992). Sleep measures. Measuring Functioning and Well-Being The Medical Outcomes Study Approach.

[B17] Hays RD, Martin SA, Sesti AM, Spritzer KL (2005). Psychometric properties of the medical outcomes study sleep measure. Sleep Med.

[B18] Guy W (1976). Clinical Global Impression (CGI). ECDEU Assessment Manual for Psychopharmacology.

[B19] Abetz L, Arbuckle R, Allen R, Garcia-Borreguero D, Hening W, Walters A, Mavraki E, Kirsch J (2004). Validating the International Restless Legs Scale (IRLS) in a trial patient population.

[B20] Campbell DT, Fiske DW (1959). Convergent and discriminant validation by the multitrait-multimethod matrix. Psychol Bull.

[B21] Bennett RM, Schein J, Kosinski MR, Hewitt DJ, Jordan DM, Rosenthal NR (2005). Impact of fibromyalgia pain on health-related quality of life before and after treatment with tramadol/acetaminophen. Arthritis Rheum.

[B22] Kazis LE, Anderson JJ, Meenan RF (1989). Effect Sizes for interpreting changes in health Status. Med Care.

[B23] Cohen J (1988). Statistical Power Analysis for the Behavioral Sciences.

[B24] Guyatt GH, Osaba D, Wu AW, Wyrwich KW, Norman GR, the Clinical Significance Consensus Meeting Group (2002). Methods to explain the clinical significance of health status measures. Mayo Clin Proc.

[B25] Dunteman GH (1989). Principal Components Analysis Sage University Paper series on Quantitative Applications in the Social Sciences, Series No 69.

[B26] Scheitel SM, Boland BJ, Wollan PC, Silverstein MD (1996). Patient-physician agreement about medical diagnosis and cardiovascular risk factors in the ambulatory general medication examination. Mayo Clin Proc.

[B27] Abetz L, Shumaker S, Marciniak A, Thorsen H, Niero M, Kohlmann T (2003). Agreement levels between clinicians' and patients' reports of health: results from an international study of postmenopausal women.

[B28] Bouchet C, Guillemin F, Briancon S (1996). Non-specific effects in longitudinal studies: impact on quality of life measures. J Clin Epidemiol.

