# Supplementary material for: The reliability, validity and responsiveness of the Restless Legs Syndrome Quality of Life questionnaire (RLSQoL) in a trial population
Source: Health Qual Life Outcomes. 2005 Dec 5;3:79. doi: 10.1186/1477-7525-3-79 (PMC1322229; doi:10.1186/1477-7525-3-79)
Supplement: Additional File 1 — Appendix: RLS Quality of Life Questionnaire [file 1477-7525-3-79-S1.doc]

## Appendix

**RLS Quality of Life Questionnaire[[1]](#endnote-2)**

**The following are some questions on how your Restless Legs Syndrome might affect your quality of life. Answer each of the items below in relation to your life experience in the past 4 weeks. Please mark only one answer for each question.**

**In the past four weeks:**

1. How distressing to you were your restless legs?

🞏 Not at all 🞏 A little 🞏 Some 🞏 Quite a bit 🞏 A lot

2. How often in the past 4 weeks did your restless legs disrupt your routine evening activities?

🞏 Never 🞏 A few times 🞏 Sometimes 🞏 Most of the time 🞏 All the time

3. How often in the past 4 weeks did restless legs keep you from attending your evening social activities?

🞏 Never 🞏 A few times 🞏 Sometimes 🞏 Most of the time 🞏 All the time

4. In the past 4 weeks how much trouble did you have getting up in the morning due to restless legs?

🞏 None 🞏 A little 🞏 Some 🞏 Quite a bit 🞏 A lot

5. In the past 4 weeks how often were you late for work or your first appointments of the day due to restless legs?

🞏 Never 🞏 A few times 🞏 Sometimes 🞏 Most of the time 🞏 All the time

6. How many days in the past 4 weeks were you late for work or your first appointments of the day due to restless legs?

Write in number of days: 

7. How often in the past 4 weeks did you have trouble concentrating in the afternoon?

🞏 Never 🞏 A few times 🞏 Sometimes 🞏 Most of the time 🞏 All the time

8. How often in the past 4 weeks did you have trouble concentrating in the evening?

🞏 Never 🞏 A few times 🞏 Sometimes 🞏 Most of the time 🞏 All the time

9. In the past 4 weeks how much was your ability to make good decisions affected by sleep problems?

🞏 None 🞏 A little 🞏 Some 🞏 Quite a bit 🞏 A lot

10. How often in the past 4 weeks would you have avoided traveling when the trip would have lasted more than two hours?

🞏 Never 🞏 A few times 🞏 Sometimes 🞏 Most of the time 🞏 All the time

11. In the past 4 weeks how much interest did you have in sexual activity?

🞏 None 🞏 A little 🞏 Some 🞏 Quite a bit 🞏 A lot

🞏 Prefer not to answer

12. How much did restless legs disturb or reduce your sexual activities?

🞏 None 🞏 A little 🞏 Some 🞏 Quite a bit 🞏 A lot

🞏 Prefer not to answer

13. In the past 4 weeks how much did your restless legs disturb your ability to carry out your daily activities, for example carrying out a satisfactory family, home, social, school or work life?

🞏 Not at all 🞏 A little 🞏 Some 🞏 Quite a bit 🞏 A lot

14. Do you currently work full or part time (paid work, unpaid or volunteer)?
 (mark one box)

🞏 YES If Yes please answer questions #15 through #18
🞏 NO, because of my RLS – Please go to the next page

🞏 NO, due to other reasons – Please go to the next page

15. How often did restless legs make it difficult for you to work a full day in the past 4 weeks?

🞏 Never 🞏 A few times 🞏 Sometimes 🞏 Most of the time 🞏 All the time

16. How many days in the past 4 weeks did you work less than you would like due to restless legs?

Write in number of days: □□

17. On the average, how many hours did you work in the past 4 weeks?

Write in number of hours per day:  □□

18. On days you worked less than you would like, on average about how many hours less did you work due to your restless legs?

Write in number of hours per day □□

## Scoring

A summary score can be calculated for the RLS quality of life questionnaire based on the following items: 1–5, 7–10 and 13. All items must be recoded such that 1 equals most severe and 5 equals least severe, so that lower scores indicate worse quality of life. The score is then transformed to a 0–100 score using the following algorithm:

[(Actual raw score – lowest possible raw score)/Possible raw score range] X 100.

If more than two items are missing from the summary scale, the summary scale score cannot be calculated and is set to missing. If one or two items from the summary scale are missing, then a person-specific estimate is substituted for that missing item. This person-specific estimate is the average score, across the completed items in the summary scale, for that respondent.

Items 6 and 16–18 are scored as continuous variables, as written by the patient. For items 6 and 16, the minimum number of days is 0 and the maximum number of days is 28. For items 17 and 18, the minimum number is 0 hours and the maximum number is 24 hours. If the response to one of these items is missing or out of range, than that item is set to missing. Items 14–18 are work-related items, thus if patients reply ”2” or ”3” to item 14, they are not expected to reply to items 15–18. Thus, the missing data rates for items 15–18 will be artificially inflated.

Items 11, 12 and 15 should be scored as categorical variables. Finally, item 14 can also be treated as a categorical variable as follows: ”yes” = 1; ”no, because of my RLS” = 2; ”no because of other reasons” = 3. If a response to one of these items is missing, then no score can be calculated for that item.

1. Copyright 2002, Richard Allen; reproduced with kind permission [↑](#endnote-ref-2)
